# High-Temperature Behavior of Laser Electrodispersion-Prepared Pd/ZSM-5 Hydrocarbon Traps under CO Oxidation Conditions

**DOI:** 10.3390/ma16124423

**Published:** 2023-06-15

**Authors:** Tatiana N. Rostovshchikova, Marina I. Shilina, Konstantin I. Maslakov, Sergey A. Gurevich, Denis A. Yavsin, Grigory B. Veselov, Vladimir O. Stoyanovskii, Aleksey A. Vedyagin

**Affiliations:** 1 Department of Chemistry, Lomonosov Moscow State University, 1/3 Leninskie Gory, 119991 Moscow, Russia; mish@kinet.chem.msu.ru (M.I.S.); nonvitas@gmail.com (K.I.M.); 2 Ioffe Physico-Technical Institute, Russian Academy of Sciences, 26 Politechnicheskaya Street, 194021 Saint Petersburg, Russia; gurevich@quantel.ioffe.ru (S.A.G.); yavsin@mail.ioffe.ru (D.A.Y.); 3 Boreskov Institute of Catalysis, 5 Lavrentyev Avenue, 630090 Novosibirsk, Russia; g.veselov@catalysis.ru (G.B.V.); stoyn@catalysis.ru (V.O.S.); vedyagin@catalysis.ru (A.A.V.)

**Keywords:** zeolite, palladium, laser electrodispersion, hydrocarbon traps, CO oxidation, prompt thermal aging

## Abstract

Zeolites and metal-doped zeolites are now widely considered as low-temperature hydrocarbon traps to be a part of emission control systems in automobiles. However, due to the high temperature of exhaust gases, the thermal stability of such sorbent materials is of great concern. To avoid the thermal instability problem, in the present work, laser electrodispersion was used to deposit Pd particles on the surface of ZSM-5 zeolite grains (SiO_2_/Al_2_O_3_ = 55 and SiO_2_/Al_2_O_3_ = 30) to obtain Pd/ZSM-5 materials with a Pd loading as low as 0.03 wt.%. The thermal stability was evaluated in a prompt thermal aging regime involving thermal treatment at temperatures up to 1000 °C in a real reaction mixture (CO, hydrocarbons, NO, an excess of O_2_, and balance N_2_) and a model mixture of the same composition with the exception of hydrocarbons. Low-temperature nitrogen adsorption and X-ray diffraction analysis were used to examine the stability of the zeolite framework. Special attention was paid to the state of Pd after thermal aging at varied temperatures. By means of transmission electron microscopy, X-ray photoelectron spectroscopy, and diffuse reflectance UV–Vis spectroscopy, it was shown that palladium, having been initially located on the surface of zeolite, undergoes oxidation and migrates into the zeolite’s channels. This enhances the trapping of hydrocarbons and their subsequent oxidation at lower temperatures.

## 1. Introduction

In order to decrease the negative impact of automobile exhaust gases on the environment, most modern vehicles are equipped with emission control systems. In the case of gasoline-powered vehicles, the main component of the emission control system is a three-way catalytic converter capable of simultaneously oxidizing CO and hydrocarbons and reducing NO_x_. Three-way catalysts are usually represented by cordierite monolith of the honeycomb structure with a deposited wash coat consisting of alumina-based material with supported precious metals such as Pd, Pt, and Rh [1,2,3,4]. However, the catalyst can reach the operating mode only when the temperature is sufficiently high. The essence of the so-called “cold-start problem” lies in the fact that at low temperatures, hydrocarbons, CO, and NO_x_ pass through the catalytic converter without reacting. Indeed, it was shown that, despite the short period of cold start emissions, they account for a significant portion of the CO and hydrocarbon total emissions [5,6,7]. To solve this problem, a number of adsorbent materials have been developed to trap the hydrocarbons and NO_x_ at low temperatures. Among them, zeolites and silicaalumophosphates are the most notable [8,9,10,11,12,13].

Zeolites possess rather high thermal stability, a large amount of surface acidic sites, a high specific surface area, and a unique microporous structure. Various types of zeolite structures have been investigated for cold start application [8,14,15,16,17,18]. According to Westermann and Azambre [10], MOR (mordenite) and MFI (Mobil Five) structures appear to be less selective for any type of hydrocarbon. Therefore, they are the most suitable to solve the cold start problem. On the other hand, it is known that the SiO_2_/Al_2_O_3_ ratio has a notable effect on the adsorption properties and stability of the zeolite. According to Burke et al. [19], in the case of BEA (beta) zeolite, the higher SiO_2_/Al_2_O_3_ ratios correspond to lower adsorption capacity in dry conditions, whereas in the presence of water, an opposite tendency is observed. The same effect was observed for the Ag-doped ZSM-5 systems [20]. Doping the zeolite with metal ions such as Ag^2+^, Fe^2+^, and Cu^2+^ was shown to increase the adsorption capacity toward hydrocarbons significantly [17,21,22,23,24,25,26]. This is due to the modification of acidic sites with metal ions. For NO_x_ capture, systems based on zeolites modified with precious metals such as palladium are usually applied [27,28,29,30]. Isolated Pd^2+^ ions were revealed to be the active site for NO_x_ adsorption, while the formation of PdO particles decreases the adsorption capacity [28].

It is worth noting that the high efficiency of the application of hydrocarbon and NO_x_ traps can be achieved only when the adsorbent material retains its properties while being exposed to long-term high temperatures. In addition to the stability of the structure of the zeolite framework, the state of the deposited metal also plays an important role. Thus, for Pd/ZSM/MCM-48 catalysts studied by He et al. [31,32], palladium, which was originally in a metal state, almost completely converted to an oxidized state after catalytic tests in the oxidation of benzene at temperatures up to 300 °C. According to extended X-ray absorption fine structure (EXAFS) spectroscopy, oxidation of Pd particles with oxygen already occurs at temperatures of 300–400 °C, and at temperatures of 400–500 °C, they undergo redispersion with the formation of smaller PdO particles and isolated Pd^2+^ ions [33,34]. Luo et al. [35] observed a similar effect for methane oxidation under lean conditions on the Pd/ZSM-5 system. It was shown for Pd/H-ZSM catalysts that redispersion of palladium occurs at lower temperatures than in the case of Pd/Na-ZSM. In contrast, Petrov et al. [36] reported for the Pd/ZSM-5 catalyst tested in the methane/oxygen mixture that the PdO particles in the range of 1–2 nm to 5–10 nm are already sintered at a temperature of 360 °C, while the calcination temperature at the catalyst preparation stage was as high as 600 °C. The presence of water vapors accelerates the sintering process substantially. It should be mentioned that Lewis acid sites present on the surface of zeolites play a decisive role in the stabilization of dispersed forms of PdO_x_ [37]. Thus, in the case of Pd/BEA, two active Pd^2+^ species stabilized on ion-exchanged sites of zeolites were detected: Z^−^-Pd^2+^-Z^−^ and Z^−^-Pd(OH)^+^. As reported by Lee et al. [28], treatment of the zeolite catalyst Pd/ZSM-5 with air at 750 °C results in a complete transition of PdO to isolated Pd^2+^ ions. Another important feature of metal particles deposited on the surface of zeolites is that they can migrate into the zeolite’s channels at elevated temperatures and modify the acidic sites. Usually, such modification enhances the adsorption capacity of zeolites toward hydrocarbons due to the presence of metal in the ionic state. For the first time, Starokon et al. [38] reported the effect of zeolite modification under prompt thermal aging conditions for the Fe/ZSM-5 system. The abnormal CO oxidation light-off curves observed in this work were explained by enhanced adsorption of hydrocarbons and their subsequent partial oxidation to CO. Further, a similar effect was reported by Temerev et al. [39] for Ag-modified ZSM-5 zeolite. It should be noted that the prompt thermal aging (PTA) mode used in these studies was allowed following the catalytic performance of the samples during a stepwise increase in the aging temperature.

Palladium-containing catalysts, where Pd is supported on aluminum oxide [40,41] or zeolites [42,43,44,45,46,47,48,49,50], are widely studied ones. The dispersion of palladium particles is often considered as the main factor defining the catalytic activity [51,52,53,54]. On the other hand, the metal–support interaction (MSI) also affects the activity and high-temperature behavior of various catalysts. Therefore, MSI attracts great attention of researchers as well [55,56,57,58].

In addition, taking into account the high cost of precious metals, the reduction of their concentration in catalysts seems to be one of the actual tasks. Whereas the controlled chemical synthesis of low percentage catalysts remains a challenge, one-step and size-controlled methods of nanoparticle deposition based on laser ablation in liquid have begun to be widely used in the synthesis of catalysts [59,60,61,62]. Among them, laser electrodispersion (LED) of metal [63,64] seems to be a prospective technique for preparing catalysts containing ultra-low metal concentrations. In this method, a laser attacks a metal target in a vacuum, which leads to the formation of metal drops. Their subsequent cascade fission in the laser torch plasma results in the formation of one-size nanoparticles. These particles are then deposited on the outer surface of the granulated support. The LED method was successfully applied for the preparation of monodispersed crust-like catalysts based on supports of various natures such as alumina, zeolites, and carbon for different reactions [65,66,67].

In our recent work [68], palladium particles of 2 nm in size were deposited on the surface of Al_2_O_3_ and ZSM-5 supports via the LED technique. While testing the catalytic performance of the samples, it was found that Pd also migrates from the surface of zeolite to its channels, which led to the stabilization of Pd^2+^ ions near the acid sites. As expected, the modification of these sites with palladium increased their sorption capacity toward hydrocarbons, and the corresponding sorption effect appeared in the CO conversion light-off curves.

The aim of the present research was an in-depth study of the high-temperature behavior of the LED-prepared Pd/ZSM-5 samples. The samples were subjected to thermal aging at temperatures up to 1000 °C in the two reaction mixtures called “model” and “real”. The model mixture contains oxygen, CO, and NO only, whereas hydrocarbons (methane, propylene, and toluene) were present in the case of the real mixture. The profile of the catalytic tests corresponded to the PTA mode [39,68]. The effect of the SiO_2_/Al_2_O_3_ ratio on the catalytic performance of Pd-modified ZSM-5 zeolites was studied as well. The stability of the zeolite framework was evaluated via X-ray diffraction analysis and low-temperature nitrogen adsorption. The samples were examined in an ethane hydrogenolysis testing reaction, which allowed comparing the surface concentration of active Pd species. The state of palladium before and after PTA tests was investigated via transmission electron microscopy, X-ray photoelectron spectroscopy, and diffuse reflectance UV–Vis spectroscopy.

## 2. Materials and Methods

### 2.1. Preparation of the Samples

HZSM-5 zeolite was obtained via calcination of the ammonia form of ZSM-5 with SiO_2_/Al_2_O_3_ ratios of 30 and 55 (Zeolyst Int., Conshohocken, PA, USA) at 550 °C for 8 h. The granule size of these zeolites was 0.4–0.8 mm. The deposition of Pd nanoparticles on the supports was carried out via laser electrodispersion (LED) as described elsewhere [64,66,68]. The bulk Pd target was exposed to radiation of a pulsed YAG:Nd laser (wavelength 1.06 μm, pulse duration 30 ns, pulse energy 120 mJ) in a vacuum. Support granules were placed in a special vibrating cell, which provided an intense stirring of granules and ensured uniform covering of their surface with Pd particles. In accordance with the calibration obtained previously [64], the deposition time of 150 μg of Pd per 0.5 g of the support was 4 min. The obtained samples were denoted as Pd/Z30 and Pd/Z55, where numbers (30 and 55) indicate the SiO_2_/Al_2_O_3_ ratio. According to the data of atomic absorption spectroscopy (Thermo Fisher Scientific Inc., Waltham, MA, USA), the Pd content in the Pd/Z30 and Pd/Z55 samples was 0.030 ± 0.002 wt.%.

For diffuse reflectance UV–Vis studies, two reference samples containing 0.2 wt.% Pd were prepared by an incipient wetness impregnation of γ-Al_2_O_3_ with the K_2_[Pd(NO_2_)_4_] solution. The first sample was dried at 105 °C for 6 h and calcined in air at 550 °C for 1 h at a heating rate of 10 °C/min. The second sample, after drying, was calcined in a 5 vol.% H_2_/Ar mixture at 300 °C for 1 h at a heating rate of 10 °C/min. These reference samples contain dispersed surface Pd^2+^ species and highly dispersed Pd^0^ particles, respectively. Therefore, these samples were denoted as Pd^2+^/A-Imp and Pd^0^/A-Imp.

### 2.2. Testing the Catalytic Performance

The high-temperature behavior of the Pd/ZSM-5 samples was examined in the PTA mode [39,68]. To follow the changes in activity and state of palladium species, a CO oxidation reaction was used. This reaction is widely used as a model reaction in the fundamental research of heterogeneous catalytic processes [69,70,71,72,73]. The samples were tested in 11 heating/cooling runs in the two reaction mixtures, which differed in the presence of hydrocarbons. The PTA mode implies an increase in the final temperature of each second catalytic run. The final temperature of each run and the compositions of the reaction mixtures are summarized in Tables S1 and S2 (see Supplementary Materials). The specimen of the sample was crashed to the fraction of 0.25–0.5 mm (300 mg) and loaded into a flow-through quartz reactor. The flow rate of the reaction mixture was 334 mL/min. The concentration of CO at the reactor outlet was monitored using a ULTRAMAT 6 gas analyzer (Siemens, Munich, Germany). The 50% conversion of CO (T_50_) was used as a criterion to compare the samples. The accuracy in the determination of this parameter was ±1 °C.

It should be emphasized that after testing, the color gradient of the sample in the reactor was observed. Therefore, in order to study the portions of the sample located in different areas of the catalyst bed, layer-by-layer loading was used. The sample was placed in the reactor in three layers (100 mg) separated by quartz wool. The catalytic activity of the individual layers was compared under the model mixture conditions at a load of 90 mg.

To reveal the state of palladium after aging at various temperatures, the samples after the sixth, eighth, and tenth runs were collected. These samples were additionally labeled with suffixes such as PTA800, PTA900, and PTA1000.

### 2.3. Characterization of the Samples

Nitrogen adsorption/desorption isotherms were recorded at 77 K using a Sync200 instrument (3P Instruments GmbH & Co. KG, Odelzhausen, Germany). To calculate specific surface area (SSA), the Brunauer–Emmett–Teller (BET) method was used. Total pore volume (V_total_) was determined from the maximum adsorption value at P/P_0_ = 0.995. Characteristics such as the volume of micropores (V_μ_) and internal (S_int_) and external (S_ext_) surface areas were determined via the t-plot (the De Boer method) [74]. This method is commonly used to analyze the microporosity of zeolites [75,76].

X-ray diffraction (XRD) analysis of the samples was performed using an automatic powder diffractometer STADI-P (Stoe GmbH, Darmstadt, Germany) installed in Bragg–Brentano geometry. CuK_α_ radiation with a wavelength of λ = 1.54060 Å was used. The XRD patterns were recorded in a 2θ range of 5–80 degrees.

Transmission electron microscopy (TEM) images were obtained using a JEM 2100F/UHR instrument (JEOL, Tokyo, Japan) working with a resolution of 0.2 nm and a maximum magnification of ×10^6^ times. The microscope is equipped with an EDX accessory. The samples were prepared for TEM studies as described elsewhere [66,68].

The samples were analyzed by X-ray photoelectron spectroscopy (XPS) on an Axis Ultra DLD spectrometer (Kratos Analytical, Manchester, UK) using a monochromatic Al*K_α_* radiation source (*hν* = 1486.7 eV; 150 W) with the pass energies of 160 eV and 40 eV. Zeolite pellets were analyzed without preliminary grinding. The Kratos charge neutralizer system was used, and the spectra were charge-corrected to give the Si2p peak a binding energy of 103.6 eV, typical for SiO_2_. Pd3d spectra were fitted with three synthetic components as detailed in Section S1.2 (Supplementary Materials). Parameters of peaks constrained into synthetic Pd3d XPS components of Pd^0^, PdO, and Pd^2+^ species are presented in Table S3.

To register diffuse reflectance UV–Vis spectra in a range from 190 to 800 nm, a UV–Vis spectrometer Varian Cary 300 UV/VIS Bio (Agilent Technologies, Inc., Santa Clara, CA, USA) was used. The spectrometer is equipped with DRA-CA-3300 (Varian, Inc., Palo Alto, CA, USA) integrating sphere with Spectralon^®^ standard material as a reference. The UV–Vis spectra were transformed into the Kubelka–Munk function (Equation (1)) [77]:

F(R) = *α/s* (1)

where *α* is the absorption, and *s* is the scattering.

The Pd-modified and pure zeolite samples were examined in a naturally hydrated state under atmospheric conditions, previously ground in an agate mortar to a uniform state.

The ethane hydrogenolysis testing reaction was applied for additional characterization of supported palladium species. The specimen (100 mg; a fraction of 0.25–0.5 mm) was loaded into a flow-through quartz reactor. Helium flow was mixed with hydrogen, and the H_2_/He mixture was passed through the reactor. The reactor was heated to 200 °C and maintained at such conditions until the system reached a stationary state. Then, ethane was added to the gas mixture for 3 min. Thereafter, a sample of the mixture at the reactor outlet was taken for analysis, which was performed using a Crystal-2000M chromatograph (Chromatec Instruments, Yoshkar-Ola, Russia) with a flame ionization detector. Then, the reactor was purged again with the H_2_/He mixture for 10 min to recover the initial state of the sample. At each temperature point within a range of 200–540 °C, the procedure was repeated 5 times.

## 3. Results and Discussions

### 3.1. High-Temperature Catalytic Performance of the Samples

**Figure 1:**
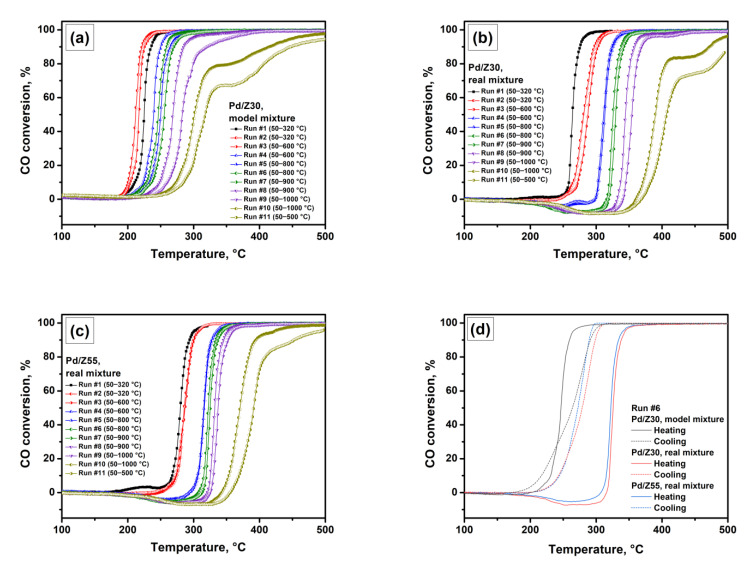
CO oxidation light-off curves recorded in the PTA mode: (**a**) Pd/Z30 sample tested in the model mixture; (**b**) Pd/Z30 sample tested in the real mixture; (**c**) Pd/Z55 sample tested in the real mixture; (**d**) Comparison of the light-off curves for heating (solid lines) and cooling (dash lines) stages of run #6.

Figure 1 shows light-off curves recorded in the PTA mode for the Pd/Z30 and Pd/Z55 samples. The corresponding T_50_ values are summarized in Table 1. Figure 1a presents the performance of the Pd/Z30 sample in the model reaction mixture. As seen after the first run with the maximum temperature of 320 °C, the second light-off curve is slightly shifted to the left. This indicates that some changes in the surface palladium species occurred during the first run. Starting from the third run, the light-off curves shift toward higher temperatures, thus indicating the deactivation of the catalyst. After aging at 900 °C, the curves become less steep at higher CO conversion values. Moreover, a noticeable plateau appears on the curves in the last two runs after the treatment at 1000 °C. As was reported by Duprat [78], internal diffusion resistance can lead to a decrease in the slope of the light-off curve, and external diffusional limitation can result in incomplete conversion and the appearance of a plateau on the curve. Probably, during the high-temperature treatment in the PTA mode, changes in the state of palladium and its localization occur, thus resulting in the appearance of diffusion restrictions.

In the case of the real reaction mixture, the catalytic performance of the Pd/Z30 sample seems to be different (Figure 1b). As seen, the presence of hydrocarbons affects the high-temperature behavior noticeably. Two main differences can be distinguished from the experiment conducted in the model mixture. First, under the real mixture conditions, the CO conversion curves are shifted to the high-temperature region. This is primarily due to the phenomenon of competitive sorption of CO and hydrocarbons on the same active sites. A decrease in the degree of palladium coverage with CO molecules reduces the rate of the CO oxidation reaction. Second, negative values of CO conversion are observed in the temperature region of 200–350 °C, thus indicating the release of an odd amount of CO. It appears that hydrocarbons adsorbed on the acidic sites of the zeolite are being partially oxidized within this temperature range, resulting in the formation of additional CO. In the case of Fe/ZSM-5 catalysts studied at the same conditions, the reactivity of CO and hydrocarbons with respect to oxygen was ranked as C_3_H_6_ > CO > C_6_H_5_CH_3_ > CH_4_ [38]. Due to the irreversible adsorption of propylene and toluene, their oxidation is enhanced. During the PTA runs, the curves are shifted to the high-temperature region, which is accompanied by the expansion of the temperature range of negative CO conversion. This indicates the maintenance of the strength of the acidic sites of the zeolite during the heat treatment.

The same character of the light-off curves is observed for the Pd/Z55 sample (Figure 1c). Among the differences, one can distinguish slightly less activity in the first runs, with greater thermal stability, which is especially noticeable in the CO conversion curves in runs #10 and #11. At the same time, at the cooling stage, the T_50_ values for the Pd/Z30 sample are lower than that for the Pd/Z55 sample. These samples are probably characterized by slightly different palladium states, which affect the nature of reversible processes occurring during the heating/cooling cycle.

Figure 1d compares the heating and cooling curves in run #6. As seen for the Pd/Z30 and Pd/Z55 samples tested in the real mixture, the shapes of the curves coincide with each other. The cooling curve is shifted by about 50 °C to the lower-temperature region and is close to the S-shape. Note that in run #11, this shift reaches 90 °C. When tested in the model mixture, a reverse temperature hysteresis phenomenon was observed for the Pd/Z30 sample. In this case, the drop in activity begins at higher temperatures during the cooling stage than during the heating stage. Moreover, in the case of the model mixture, the differences in T_50_ values during the heating and cooling stages do not exceed 30 °C. Such differences can be explained by the two factors. First, it is the phenomenon of competitive sorption of CO and hydrocarbons, which significantly changes the kinetics of CO oxidation. Second, the oxidation of toluene and propylene results in the release of additional amounts of CO.

**Figure 2:**
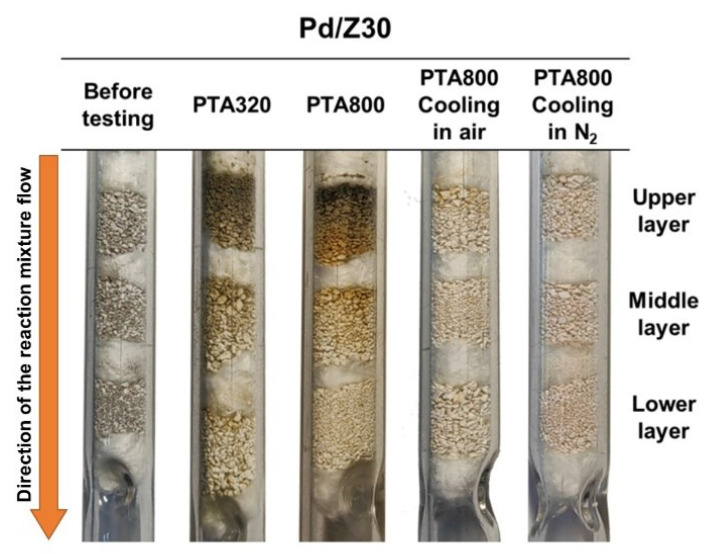
Photographs of the quartz reactor with the Pd/Z30 sample at different stages of the experiment.

During the experiments, it was found that the sample changed color and became darker. The most intense darkening is observed for the part of the sample that is primarily exposed to the reaction medium. In order to characterize different parts of the sample by physicochemical methods, layer-by-layer loading experiments were carried out. Three layers of the sample were loaded into the reactor and separated by quartz wool. Figure 2 shows photographs of the reactor with the loaded Pd/Z30 sample before and after catalytic tests. As can be seen, the darkening of the sample can already be observed after its treatment in the real mixture at 320 °C. After thermal aging at higher temperatures, the effect becomes more noticeable. Therefore, to reveal the possible reasons for observed darkening, additional experiments were carried out. In these catalytic tests, the reaction mixture was switched to air or pure nitrogen at a temperature of 800 °C immediately after the end of the heating stage of run #6. In both cases, no darkening of the sample was observed. Thereby, darkening appears to be a consequence of the coke formation during the cooling step at low temperatures.

**Figure 3:**
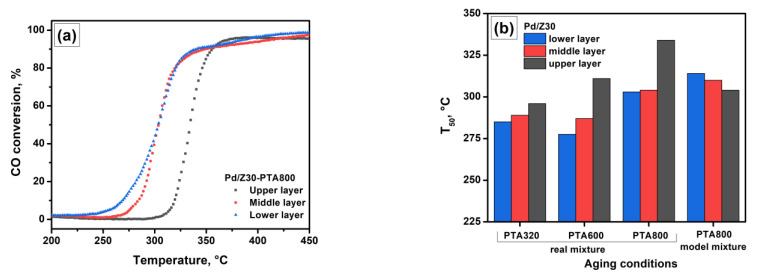
CO oxidation light-off curves over the upper, middle, and lower layers of the Pd/Z30 sample after PTA800 (**a**). T_50_ values for the various layers of the Pd/Z30 sample after PTA at different conditions (**b**).

It is obvious that the state of the sample is different in different areas of the catalyst bed. Therefore, the catalytic performance of separate layers was studied. First, the Pd/Z30 sample was loaded into the reactor in a layer-by-layer mode (three layers separated by quartz wool) and subjected to the PTA treatment. Then, the CO oxidation activity of each layer was tested in the model mixture. Figure 3a shows the light-off curves for the upper, middle, and lower layers of the Pd/Z30-PTA800 sample. It can be seen that the CO conversion curve for the upper layer is shifted to the region of higher temperatures. The catalytic performance of the lower and middle layers are close to each other, although, in the case of the lower layer, conversion begins at lower temperatures. It seems that the presence of carbon deposits in the upper layer leads to partial blockage of palladium particles, which leads to an increase in the light-off temperature. When the temperature increases, carbon deposits are removed due to their oxidation with oxygen contained in the reaction mixture. Figure 3b compares the catalytic performance of three layers after aging in the real mixture at 320, 600, and 800 °C, as well as after aging at 800 °C in the model mixture. Even after the treatment at 320 °C, the layers can be ranked as follows: upper layer < middle layer < lower layer. Contrarily, an inverse row is observed under the model mixture conditions, although the T_50_ values for the three layers are quite close. While summarizing, it can be said that although the presence of hydrocarbons negatively affects the catalytic performance of the samples due to the formation of carbon deposits, only the upper layer of the catalyst is influenced noticeably.

### 3.2. Characterization of the Samples at Various Stages of the Catalytic Tests

**Figure 4:**
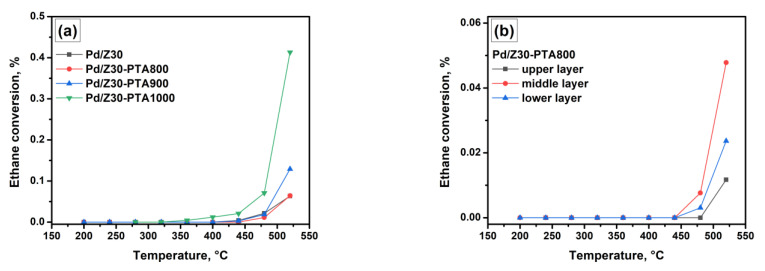
Ethane hydrogenolysis over the Pd/Z30 sample: (**a**) comparison of the initial and PTA-aged samples; (**b**) comparison of the three layers.

As was reported by Yates and Sinfelt [79,80], the ethane hydrogenolysis reaction is applicable for assessing the surface concentration of supported precious metals (Pd and Rh) in the reduced state. However, this technique has not been used to test zeolite-based systems yet. Figure 4a demonstrates the curves of ethane conversion over the Pd/Z30 sample treated in the PTA mode under the real mixture conditions in comparison with the as-prepared sample. Note that, for these tests, the sample was blended thoroughly, and 100 mg was sampled. Therefore, the data presented in Figure 4a reflect the average state of the samples.

No significant changes can be observed when comparing the as-prepared sample with the PTA-aged Pd/Z30-PTA800 sample. Then, the ethane conversion grows in a row Pd/Z30-PTA800 < Pd/Z30-PTA900 < Pd/Z30-PTA1000. On the one hand, an increase in activity in the ethane hydrogenolysis reaction should be a consequence of an increase in the dispersion of Pd species. However, this does not agree with the data on catalytic performance in CO oxidation, where an opposite row is observed.

It is worth noting, however, that hydrogenolysis on isolated metal ions or small clusters can be complicated since several adjacent metal atoms are required to convert one molecule of ethane. For example, Yates and Sinfelt [79,80] observed a decrease in the specific catalytic activity of the Rh/SiO_2_ catalyst in the ethane hydrogenolysis reaction with a decrease in rhodium concentration to 0.1–0.3% that corresponds to the crystallite size less than 1.2 nm. On the other hand, in the case of zeolites used as a support, an acceleration of the hydrogenolysis reaction due to the presence of acid sites near Pd species, which can participate in the activation of the ethane molecule, cannot be ruled out. The results of the PTA tests allow us to conclude that the acid sites remain even after aging at 1000 °C. Taking into account the previously discussed literature data, it can be assumed that palladium migrates into the channels of the ZSM-5 zeolite under PTA conditions. This process is accompanied by the oxidation of palladium particles with the formation of dispersed PdO species and isolated Pd^2+^ ions. The latter seems to be less active in the CO oxidation reaction but yields smaller Pd^0^ particles with higher Pd specific surface area after reduction and, therefore, higher activity in ethane hydrogenolysis.

The ethane conversion curves for the three layers of the Pd/Z30-PTA800 sample are compared in Figure 4b. As seen, the lowest activity in the ethane hydrogenolysis reaction was exhibited by the upper layer. It can be assumed that in the upper layer, which first contacts with the reaction mixture, there was a coarsening of Pd^0^ particles compared to the as-prepared sample. It should also be noted that carbon can react with hydrogen according to the following reaction: C + 2H_2_ ↔ CH_4_ [81,82,83,84,85]. Due to the presence of hydrogen in the reactive mixture during the ethane hydrogenolysis experiments, this process provided the removal of the carbon deposits from the catalyst surface. For the remaining part of the sample, oxidation of Pd^0^ particles and the formation of more dispersed oxidized species such as PdO and Pd^2+^ are likely to occur. In order to support all the assumptions made, a detailed characterization of the samples by physicochemical methods was carried out.

First, the textural properties of the as-prepared and PTA-aged samples calculated from low-temperature nitrogen adsorption/desorption isotherms are compared in Table 2. As an example, Figure S1 (Supplementary Information) presents the isotherms for the Pd/Z30 and Pd/Z30-PTA1000 samples. The sample of the ZSM-5 zeolite used in this research consists of crystallites of several tens of nanometers in size. Therefore, the close arrangement of these crystallites leads to the difference between the V_total_ and V_μ_ values. The shapes of the isotherms and calculated values of SSA and S_int_ (Table 2) for the as-prepared and PTA-aged samples are practically the same for Pd/Z30 and only slightly decreased for Pd/Z55. This indicates that the zeolite framework is stable under high-temperature aging conditions. Furthermore, the micropore volume (V_μ_) changes only slightly with an increase in the aging temperature, decreases for Pd/Z30, and increases just a little for Pd/Z55. The changes in the pore volume between the as-prepared and PTA-aged samples slightly exceed 9%.

The thermal stability of the zeolite framework was also confirmed by XRD analysis. The XRD patterns for the Pd/Z30 and Pd/Z30-PTA1000 samples are given in Figure S2 (Supplementary Materials). The patterns practically coincide with each other, which indicates that the zeolite structure remained intact. The loss of crystallinity degree was estimated to be within several percent. Thus, the results of low-temperature nitrogen adsorption and XRD analysis clearly show that the structure of the zeolite ZSM-5 does not undergo significant changes during the thermal aging under the real mixture conditions at high temperatures such as 1000 °C. This is in good agreement with the results of catalytic tests, from which the preservation of the adsorption sites of the zeolite was assumed.

**Figure 5:**
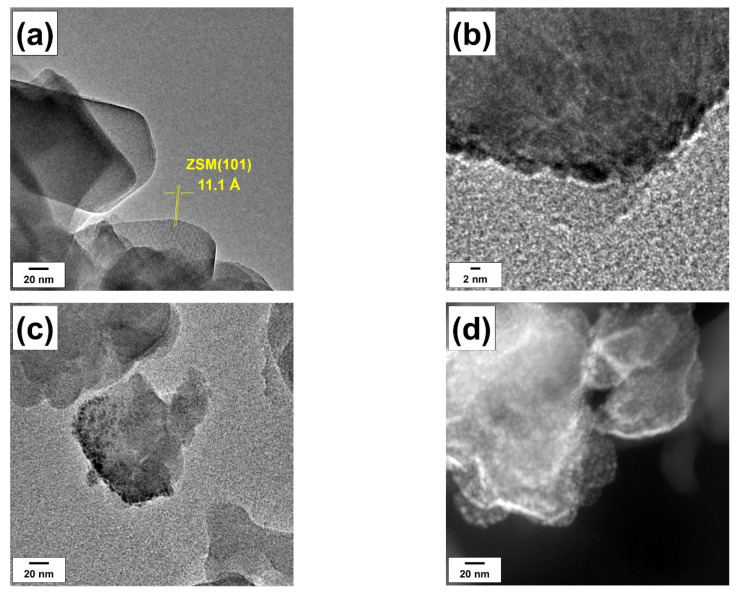
TEM images of the Z30 support (**a**) and the Pd/Z30 sample (**b**–**d**).

Transmission electron microscopy provides the possibility to analyze not only the crystal structure of the zeolite but the sizes of palladium particles as well. Figure 5 illustrates TEM images of pure Z30 support and the as-prepared Pd/Z30 sample. The zeolite matrix can be seen in Figure 5a. The interplanar distance for regions of ordered atoms on the zeolite surface is 11.1 Å, which is close to that of the ZSM (101) facet (JCPDS #29-1257, d_101_ = 11.33 Å). Typical TEM images of Pd particles are shown in Figure 5b–d. It can be seen that the Pd particles are uniformly distributed over the surface of the ZSM-5 support. They are located close to each other and form agglomerates. Their size can be estimated as about 2 nm. This is in good agreement with our previous studies, including the Pd deposition directly onto TEM grids, which showed the same 2 nm size of Pd nanoparticles regardless of the Pd content and support nature [64,66,86].

**Figure 6:**
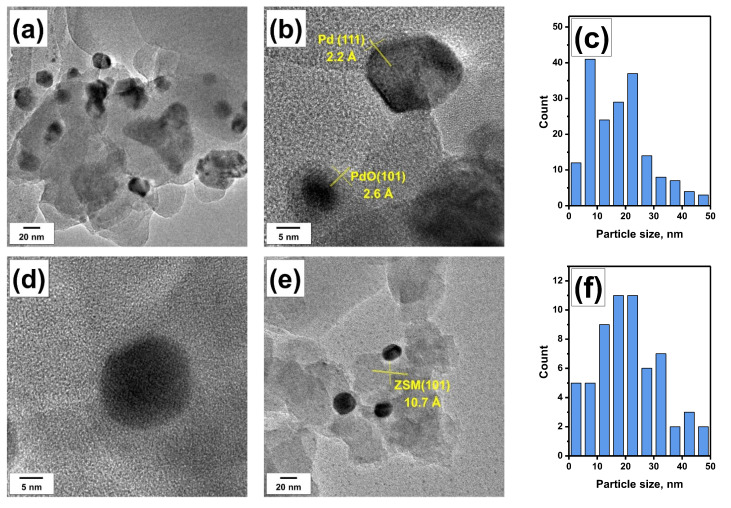
TEM images of the Pd/Z30-PTA800 sample and corresponding particle size distributions: (**a**–**c**) upper layer; (**d**–**f**) lower layer.

Figure 6 shows TEM images for the upper and lower layers of the Pd/Z30-PTA800 sample. In both cases, there was a significant coarsening of palladium particles, which is clearly seen from the particle size distributions presented in Figure 6c,f. Such sintering results in the formation of particles of up to 50 nm in size. The upper layer of the sample is characterized by the presence of relatively small particles of 5–10 nm in size. As a result, the average size of the palladium particles in the upper layer is 16 nm, which is slightly lower than in the lower layer (24 nm). Analysis of interplanar distances (Figure 6b) showed that both metal Pd^0^ and PdO particles of smaller size are present in the upper layer. It is worth noting that the coke formation in the upper layer was not observed by the TEM technique. In addition, no significant changes in the state of the ZSM-5 support were found.

When the samples were aged at a higher temperature, no palladium particles were found by TEM. Corresponding images for the Pd/Z30-PTA900, Pd/Z30-PTA1000, and Pd/Z55-PTA1000 samples are shown in Figure S3 (Supplementary Materials).

**Figure 7:**
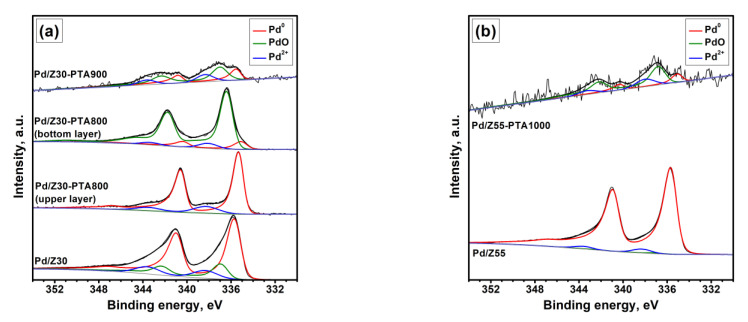
XPS spectra (Pd3d region) of the as-prepared and PTA-aged samples: (**a**) Pd/Z30; (**b**) Pd/Z55.

The XPS spectra of the as-prepared and PTA-aged Pd/Z samples are shown in Figure 7. Unusually high ratios of [Pd]/[Al] and [Pd]/[Si] calculated from XPS data (Table 3) in both the as-prepared samples confirm the crust-like nature of the Pd coating deposited by the LED method. To identify the contribution of different forms of palladium, the Pd3d spectra were fitted with three synthetic components. Along with the components of metallic palladium and palladium oxide (PdO), an additional doublet peak of Pd3d_5/2_ with a binding energy of 338.0–338.4 eV was used. It can be attributed to Pd(OH)_2_, palladium salts, or oxidized Pd^2+^ species coordinated with the support oxygen. It is worth noting that the Pd3d_5/2_ binding energy of the Pd^0^ component is slightly higher than that for bulk metallic palladium, similar to the LED-prepared Pd/Al_2_O_3_ catalysts [68]. The fitting results (Table 3) point to the mainly metallic state of palladium in both the as-prepared Pd/Z sample. However, the fraction of oxidized Pd species also exists on the surface of Pd/Z30 (about 30% in total). This is probably because of the interaction of palladium with the acid sites of the zeolite with a lower SiO_2_/Al_2_O_3_ ratio, as it was reported previously [66].

As can be seen from Table 3, after PTA tests, the electronic state and Pd content on the surface of the samples changes noticeably. In the case of the Pd/Z30-PTA800 sample (upper layer), the percentage of metallic palladium becomes even higher than in the as-prepared sample. Probably, the coke formed in the upper layer contributed to the stabilization of palladium in the reduced state. In contrast, in the Pd/Z30-PTA800 sample (lower layer), 88% of palladium is converted to PdO and Pd^2+^ species. The Pd state on the surface of Pd/Z30-PTA900 is intermediate between the upper and lower layers in the Pd/Z30-PTA800 sample.

At the same time, the [Pd]/[Al] and [Pd]/[Si] ratios decreased in both Pd/Z samples after PTA; this result confirms the abovementioned assumption that thermal aging facilitates migration of palladium into the micropores of zeolite, which makes Pd undetectable by the XPS analysis. The more pronounced decrease in Pd content observed for the upper layer in comparison with the lower one may be associated with the coke formation.

The intensity of the Pd3d signal from the Pd/Z55-PTA1000 sample was very low (Figure 7b). Even a long-term acquisition of this spectrum resulted in a noisy Pd3d peak. Apparently, in this sample, almost all palladium was located in the zeolite channels. A very low palladium content on the surface of Pd/Z30-PTA1000 made it difficult for the unambiguous fitting of its very noisy Pd3d spectrum. Nevertheless, most of the palladium in this sample is in the oxidized state.

**Figure 8:**
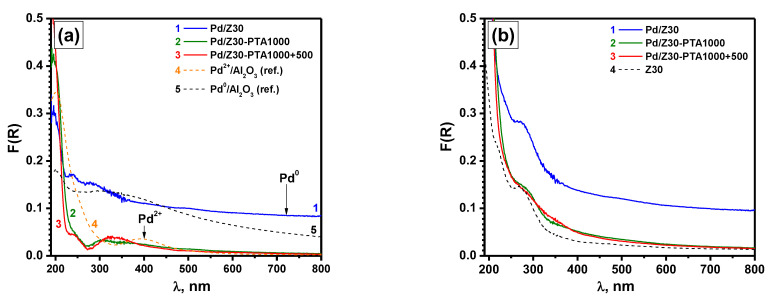
Diffuse reflectance UV–Vis spectra of the as-prepared and PTA-aged Pd/Z30 samples: (**a**) spectra after subtraction of the spectrum of pure zeolite; (**b**) original spectra. Spectra of the reference Pd^2+^/A-Imp and Pd^0^/A-Imp samples (**a**) and pure zeolite (**b**) are given for comparison.

At the last stage of the research, in order to characterize the average state of palladium in the volume of the samples, the diffuse reflectance UV–Vis spectroscopy method was applied. Figure 8a shows the corresponding spectra of the as-prepared Pd/Z30 and PTA-aged Pd/Z30-PTA1000 samples. Note that the spectrum of pure zeolite was subtracted from these spectra. The original spectra are shown in Figure 8b. In order to remove coke and adsorbed organic compounds, the Pd/Z30-PTA1000 sample was additionally calcined at 500 °C in the air (labeled as Pd/Z30-PTA1000+500). The spectra of reference samples, Pd^2+^/A-Imp containing palladium in the form of isolated Pd^2+^ ions and small PdO clusters and Pd^0^/A-Imp containing palladium in the form of dispersed metal particles, are also given for comparison. In the spectrum of the as-prepared Pd/Z30 sample (spectrum 1), a background rise in the long-wave region characteristic of metal Pd^0^ particles is observed. The spectra of the PTA-aged samples, Pd/Z30-PTA1000 (spectrum 2) and Pd/Z30-PTA1000+500 (spectrum 3), practically coincide with each other. However, no d-d transition bands for Pd^2+^ in the form of oxide clusters, isolated ions, and PdO particles are observed [87]. The samples show no absorption in the red region, which indicates complete oxidation of palladium as a result of the PTA treatment. The sequential changes in absorption spectra in the UV region (190–215 nm) are probably due to the rearrangement of the ZSM-5 support itself and do not correspond to the charge transfer band of Pd^2+^ complexes. Such behavior expressed in the absence of visible d-d transition bands can be associated with the migration of Pd^2+^ species into the volume of ZSM-5, which is accompanied by a further decrease in the intensity of d-d transitions and the charge transfer band due to decoration by the support.

## 4. Conclusions

In the present work, the laser electrodispersion method was used for the deposition of dispersed palladium particles of ~2 nm in size on the surface of ZSM-5 zeolite. The high-temperature behavior of such materials has been studied under conditions close to the real operating conditions of hydrocarbon traps, which are a crucial component of automotive exhaust gas neutralization systems. It was found that the presence of hydrocarbons in the reaction mixture substantially reduces the efficiency of the catalyst system in neutralizing CO. This is primarily due to the phenomenon of competitive sorption. The portion of the Pd-modified zeolite that is primarily in contact with the reaction mixture was more affected by hydrocarbons. At the cooling stage of the catalytic run, at low temperatures, carbon deposits are formed, which leads to the blockage of palladium particles. This increases the light-off temperature in the CO oxidation reaction. In addition, as determined by the XPS method, the state of palladium in different areas of the catalyst bed is different. In the upper layer, palladium is stated in a reduced form of metal Pd^0^ particles, while the rest of the sample is characterized by an oxidized state of palladium.

XRD and low-temperature nitrogen adsorption studies have shown that the structure of the ZSM-5 zeolite remains practically unchanged during high-temperature aging. According to TEM, XPS, and diffuse reflectance UV–Vis spectroscopy, palladium oxidizes and migrates to the channels of the ZSM-5 zeolite during the PTA treatment. An increase in palladium dispersion was also confirmed by the ethane hydrogenolysis testing reaction. At the same time, the catalytic activity in the CO oxidation reaction decreases successively as the temperature of the thermal aging increases. Palladium appears to be stabilized within the zeolite channels as separate ions or small PdO clusters. Such localization of palladium leads to mass transfer limitations, which decreases the efficiency of CO and hydrocarbon oxidation. It should be reminded that the main function of zeolites in the composition of exhaust gas neutralization systems is to trap hydrocarbons at low temperatures. Subsequently, the increased temperature of exhaust gases should provide desorption and complete oxidation of the trapped hydrocarbons. The catalytic experiments performed under the prompt thermal aging conditions have demonstrated that the adsorption properties of the Pd-modified ZSM-5 zeolite remained the same even after treatment at such a high temperature as 1000 °C. The findings of this research demonstrate the promise of using Pd/ZSM-5 materials prepared by the LED technique as efficient and thermally stable systems for trapping hydrocarbons when starting an automobile engine.

## Figures and Tables

**Table 1:** T_50_ values for the cooling and heating stages of the PTA runs.

Run Number	Pd/Z30, Model Mixture		Pd/Z30, Real Mixture		Pd/Z55, Real Mixture	
	Heating	Cooling	Heating	Cooling	Heating	Cooling
1	225	196	264	215	280	225
2	212	198	281	215	286	224
3	216	240	288	256	287	276
4	250	233	312	255	316	263
5	239	251	313	272	315	267
6	246	263	326	279	323	273
7	255	256	330	270	325	281
8	269	267	345	276	332	287
9	283	273	354	293	337	306
10	301	281	390	307	370	308
11	317	293	407	314	391	319

**Table 2:** Textural parameters of the Pd/Z samples before and after testing in the PTA mode.

Sample	SSA, m^2^/g	S_int_, m^2^/g	S_ext_, m^2^/g	V_total_, cm^3^/g	V_μ_, cm^3^/g
Pd/Z30	427	407	20	0.34	0.21
Pd/Z30-PTA800	392	371	21	0.32	0.21
Pd/Z30-PTA900	398	376	22	0.31	0.21
Pd/Z30-PTA1000	404	383	21	0.31	0.20
Pd/Z55	462	443	19	0.44	0.19
Pd/Z55-PTA1000	442	422	20	0.42	0.21

**Table 3:** XPS data for the as-prepared and PTA-aged Pd/Z samples.

Sample	[Pd]/[Al]	[Pd]/[Si]	Binding Energy of Pd3d_5/2_, eV			Percentages of Pd Species, %		
			Pd^0^	PdO	Pd^2+^	Pd^0^	PdO	Pd^2+^
Pd/Z30	10.2	0.57	335.8	337.0	338.4	70	18	12
Pd/Z30-PTA800 upper layer	0.4	0.03	335.3	-	338.2	87	0	13
Pd/Z30-PTA800 lower layer	0.6	0.04	335.1	336.4	338.1	12	81	7
Pd/Z30-PTA900	0.02	0.001	335.6	337.0	338.3	39	40	21
Pd/Z55	20.0	0.83	335.7	336.9	338.3	95	1	4
Pd/Z55-PTA1000	<0.01	<0.0004	335.1	336.8	338.0	21	59	20

## Data Availability

Data are contained within the article.

## Supplementary Materials

The following supporting information can be downloaded at: https://www.mdpi.com/article/10.3390/ma16124423/s1, Table S1: Reaction conditions for testing the catalytic performance; Table S2: Final temperatures in the PTA runs; Table S3: Parameters of peaks constrained into synthetic Pd3d XPS components of Pd^0^, PdO, and Pd^2+^ species; Figure S1: Nitrogen adsorption/desorption isotherms for the Pd/Z30 and Pd/Z30-PTA1000 samples; Figure S2: XRD patterns for the Pd/Z30 and Pd/Z30-PTA1000 samples; Figure S3: TEM images of the Pd/Z-PTA900, Pd/Z-PTA1000, and Pd/Z55-PTA1000 samples. Reference [88] is cited in the supplementary materials.
