# Supplementary material for: High-Temperature Behavior of Laser Electrodispersion-Prepared Pd/ZSM-5 Hydrocarbon Traps under CO Oxidation Conditions
Source: Materials (Basel). 2023 Jun 15;16(12):4423. doi: 10.3390/ma16124423 (PMC10305408; doi:10.3390/ma16124423)
Supplement: Supplementary file 1 [file materials-16-04423-s001.zip › materials-2401361-supplementary.pdf]

materials

Supplementary Information

High-Temperature Behavior of Laser Electrodispersion-Prepared Pd/ZSM-5 Hydrocarbon Traps under CO Oxidation Conditions

Tatiana N. Rostovshchikova [1,*], Marina I. Shilina [1], Konstantin I. Maslakov [1], Sergey A. Gurevich [2], Denis A. Yavsin [2], Grigory B. Veselov [3], Vladimir O. Stoyanovskii [3], Aleksey A. Vedyagin [3]

[1] Department of Chemistry, Lomonosov Moscow State University, 1/3 Leninskie Gory, 119991 Moscow, Russia
[2] Ioffe Physico-Technical Institute, Russian Academy of Sciences, 26 Politechnicheskaya Street, 194021 Saint Petersburg, Russia
[3] Boreskov Institute of Catalysis, 5 Lavrentyev Avenue, 630090 Novosibirsk, Russia
* Correspondence: t.rost50@mail.ru

1. Materials and Methods

1.1. Testing the catalytic performance

Table S1. Reaction conditions for testing the catalytic performance.

Mixture	CO, ppm	Methane, ppm	Propylene, ppm	Toluene, ppm	NO, ppm	O_2, vol%	N_2
Model	1500	-	-	-	150	14	balance
Real	1500	30	40	11	150	14	balance

Table S2. Final temperatures in the PTA runs.

Run number	Final temperature, °C	Run number	Final temperature, °C
1	320	7	900
2	320	8	900
3	600	9	1000
4	600	10	1000
5	800	11	500
6	800		

1.2. Fitting of Pd3d spectra

The spectra were fitted in the CasaXPS software. The U2 Tougaard background was used in the binding energy range between about 331 and 356 eV. The second parameter of the background cross section was manually adjusted to produce the best fit. The spectra were fitted with three synthetic components attributed to Pd^0, PdO, and Pd^{2+} species. The latter ones were species in a different environment than in PdO, such as $Pd(OH)_2$, palladium salts, or oxidized Pd^{2+} species coordinated with the support oxygen. Each component represented a set of constrained peaks (see Table S3). The synthetic component of Pd^0 species was constructed based on the preliminary acquired spectrum of pure metallic palladium. The spectrum of PdO oxide from [88] was used to construct the synthetic component of PdO species. An additional doublet of symmetric peaks was used as a synthetic component of Pd^{2+} species.

Table S3. Parameters of peaks constrained into synthetic Pd3d XPS components of Pd^0, PdO, and Pd^{2+} species.

**Synthetic component**	**Peak Name**	**Line Shape**	**Area**	**FWHM**	**Position**
Pd^0	A	LF(0.65,1.6,100,120)	Varied	(1.0, 1.4) [a)]	(335.0, 335.6)
	B	LF(0.65,1.6,100,120)	A×0.625	A×1.00	A+5.25
	C	GL(30)	A×0.0457	A×1.50	A+6.16
	D	GL(30)	A×0.0285	A×1.50	A+11.40
	E	GL(30)	A×0.163	A×4.48	A+7.67
	F	GL(30)	A×0.102	A×4.48	A+12.91
PdO	G	GL(50)	Varied	(1.4, 1.6)	(336.7, 337.0)
	H	GL(50)	G×0.619	G×1.15	G+5.34
	I	GL(30)	G×0.349	G×2.63	G+2.30
	J	GL(30)	G×0.233	G×2.63	G+7.64
	K	GL(30)	G×0.0583	G×2.12	G+8.94
	L	GL(30)	G×0.0389	G×2.12	G+14.3
Pd^{2+}	M	GL(30)	Varied	(1.8, 2.0)	(338.0, 338.5)
	N	GL(30)	M×0.667	M×1	M+5.3

[a)] Varied within a range.

2. Results and discussions

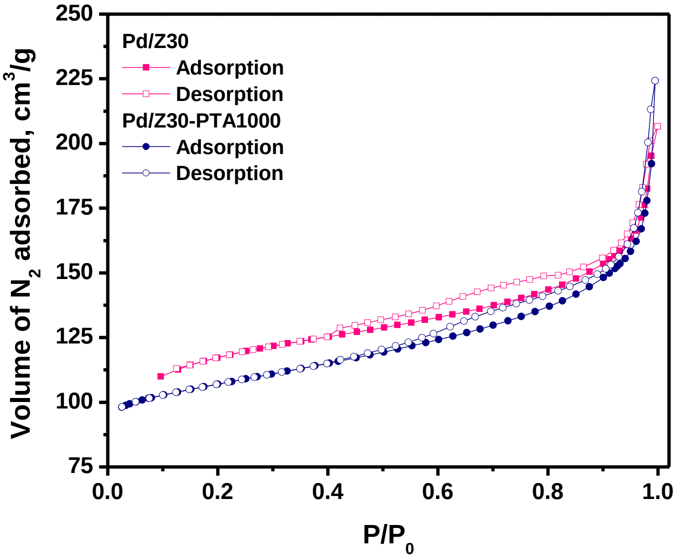

Figure S1. Nitrogen adsorption/desorption isotherms for the Pd/Z30 and Pd/Z30-PTA1000 samples.

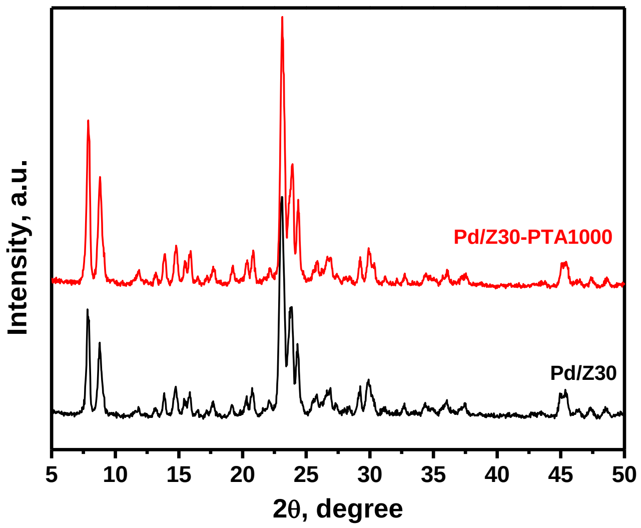

Figure S2. XRD patterns for the Pd/Z30 and Pd/Z30-PTA1000 samples.

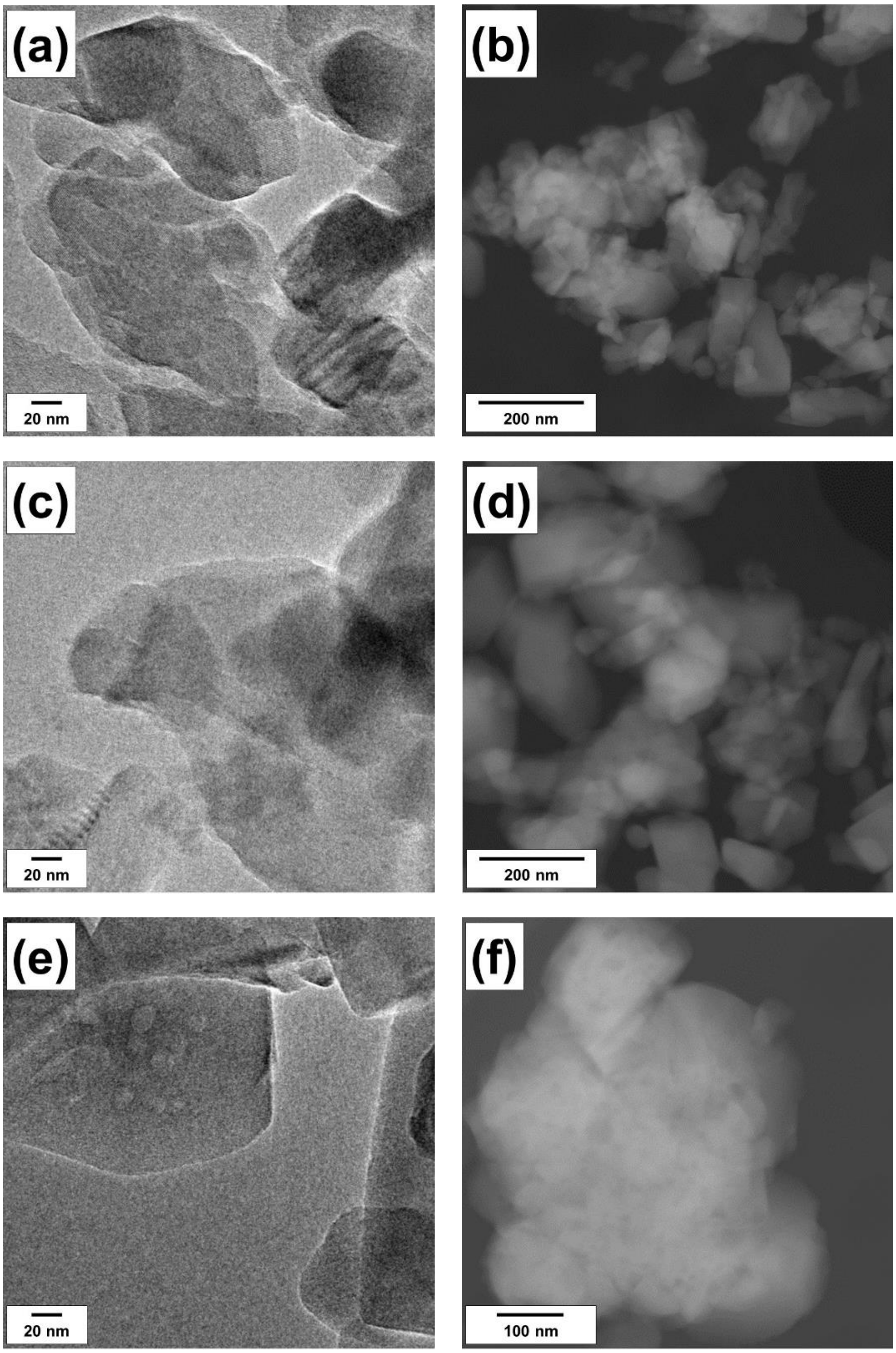

Figure S3. TEM images of the Pd/Z-PTA900 (**a, b**), Pd/Z-PTA1000 (**c, d**), and Pd/Z55-PTA1000 (**e, f**) samples.
